# The Food and Drug Administration-approved antipsychotic drug trifluoperazine, a calmodulin antagonist, inhibits viral replication through PERK-eIF2α axis

**DOI:** 10.3389/fmicb.2022.979904

**Published:** 2022-11-01

**Authors:** Yizhi Mao, Ziyang Wang, Chen Yao, Qi Zeng, Wei Cheng, Shimeng Zhang, Shuai Chen, Chunjie Sheng

**Affiliations:** ^1^State Key Laboratory of Oncology in South China, Collaborative Innovation Center for Cancer Medicine, Sun Yat-sen University Cancer Center, Guangzhou, China; ^2^Department of Gastrointestinal Surgery, The First Affiliated Hospital, Sun Yat-sen University, Guangzhou, China

**Keywords:** trifluoperazine, antiviral drugs, eIF2α, PKR-like endoplasmic reticulum kinase (PERK), calmodulin

## Abstract

Virus-related diseases are seriously threatening human health, but there are currently only 10 viruses with clinically approved antiviral drugs available. As non-cellular organisms, viruses parasitize in living cells and rely on the protein synthesis mechanism of the host cells. In this study, we found that the antipsychotic drug trifluoperazine (TFP), a dual dopamine receptor D2 (DRD2)/calmodulin (CALM) antagonist, increases the phosphorylation of eukaryotic initiation factor 2α (eIF2α), a key factor in the regulation of protein synthesis and significantly inhibits vesicular stomatitis virus (VSV) and herpes simplex virus type 1 (HSV-1) replication. CALM but not DRD2 is involved in the antiviral activity of TFP. By knockdown of protein kinase R (PKR)-like endoplasmic reticulum kinase (PERK) we found that the antiviral function of TFP is dependent on PERK, a stress response kinase that mediates eIF2α phosphorylation. Furthermore, the results of animal experiments showed that TFP protects mice from lethal VSV attacks, improving the survival rate and reducing lung injury. Taken together, these data suggests that TFP inhibits virus replication through PERK-eIF2α axis, and this broad-spectrum of mechanisms are worth further evaluation in clinical trials in the future.

## Introduction

Protein translation is a fundamental biological process. In eukaryotic cells, the process of translation has three phases, namely initiation, elongation, and termination. The initiation of translation is a complex process regulated by many factors. It can roughly be divided into several stages: formation of eukaryotic initiation factor 2 (eIF2)–GTP–Met-tRNA_i_^Met^ ternary complex (TC); formation of 43S pre-initiation complex (PIC) containing a 40S subunit, eIF1, eIF1A, eIF3, eIF5, and eIF2–GTP–Met-tRNA_i_^Met^; recruitment of PIC to the 5′ end of mRNA; scanning from the 5′ UTR by 43S PIC through anticodon in Met-tRNA_i_^Met^, identifying AUG start codon; joining of 60S subunit to PIC accompanied by a release of eIF2–GDP and other factors to form the 80S ribosome and ready for elongation (Jackson et al., 2010; Hinnebusch, 2014; Merrick and Pavitt, 2018). As a key factor involved in translation initiation, eIF2 functions to form the eIF2–GTP–Met-tRNA_i_^Met^ TC that binds to 40S subunit. eIF2 contains three subunits, α, β, and γ. Phosphorylation of eIF2α causes inhibition of global translation. During integrated stress response (ISR), phosphorylation of eIF2α leads to a decrease in protein synthesis and preferential translation of specific genes including the activating transcription factor 4 (ATF4), which help to restore protein homeostasis. The phosphorylation of eIF2α causes the inhibition of conversion from eIF2⋅GDP to the active form eIF2⋅GTP and reduces TC delivered to the ribosome. Four different kinases, heme-regulated eIF2α kinase (HRI or EIF2AK1), protein kinase R (PKR or EIF2AK2), PKR-like endoplasmic reticulum kinase (PERK or EIF2AK3) and general control non-derepressible 2 (GCN2 or EIF2AK4) mediate the phosphorylation of eIF2α on Ser51. They have extensive homology at kinase catalytic domains but different regulatory domains. Each eIF2α kinase dimerizes and autophosphorylates for activation (Pakos-Zebrucka et al., 2016; Wek, 2018).

Viruses are non-cellular organisms that must parasitize in living cells and proliferate by replication. Viruses rely on the protein synthesis mechanism of the host cells. Remarkably, nearly every step of translation can be targeted by virus-encoded function (Walsh and Mohr, 2011). Vesicular stomatitis virus (VSV) (Howatson, 1970) and herpes simplex virus type 1 (HSV-1) (Pires de Mello et al., 2016) are single negative-stranded RNA and double-stranded DNA viruses, respectively. It has been reported that the M protein secreted by VSV can promote the accumulation of hypophosphorylated 4E-BP1 by inhibiting the protein kinase B (AKT). Hypophosphorylated 4E-BP1 binds to eIF4E, blocking the combination of eIF4E and eIF4G, thus repressing eIF4F assembly and translational process (Dunn and Connor, 2011). HSV-1 can directly phosphorylate tuberous sclerosis complex 2 (TSC2) through Us3 ser/thr kinase and activate the mechanistic target of rapamycin complex 1 (mTORC1), thereby inducing 4E-BP1 phosphorylation and eIF4F assembly, promoting viral protein synthesis and replication (Chuluunbaatar et al., 2010). HSV-1 can also inhibit PKR, eIF2α phosphatase subunit and PERK through Us11, γ34.5 and viral glycoprotein B (gB), respectively, resulting in a reduction of P-eIF2α levels and promoting translation (Mulvey et al., 1999; Mulvey et al., 2003; Mulvey et al., 2007). In addition, eIF4G, eIF4A, poly(A)-binding protein (PABP), eIF2, eEF1A, and eEF1B are other targets of HSV-1 (Walsh and Mohr, 2011). The dependence of the viruses on the host protein synthesis system and the complicated interaction between viruses and the host provide new strategies for antiviral therapy. Boyce et al. (2005) reported that salubrinal, a selective inhibitor of cellular complexes that dephosphorylates eIF2α, blocks HSV protein-mediated dephosphorylation of eIF2α and inhibits virus replication. The small molecule inhibitor 4EGi-1 restrains cap-dependent translation through binding to eIF4E and thus disrupting the binding of eIF4E/eIF4G (Moerke et al., 2007) with effective non-cytotoxic antiviral effect against HSV-1 and Vaccina virus (VacV) (McMahon et al., 2011). Another study reported MAPK interacting kinase 1 (Mnk-1) inhibitor CGP57380 inhibits eIF4E phosphorylation and reduces HSV-1 replication (Walsh and Mohr, 2004).

Ca^2+^ binding protein calmodulin (CALM) is ubiquitous in eukaryotic cells and involves in various cell signal transduction pathways. There are three paralogous genes encoding calmodulin protein in the mammalian genome, named *CALM1*, *CALM2*, and *CALM3*. CALM plays a key role in Ca^2+^-dependent signal transduction pathways. CALM regulates downstream target enzymes such as cyclic nucleotide phosphodiesterase, adenylate cyclase, and myosin kinase, participates in regulating cell proliferation and cell cycle, regulates microtubule depolymerization, cell motility and Ca^2+^ fluxes (Klee et al., 1980). To find effective antiviral drugs, we did drug screening and found that Food and Drug Administration (FDA) approved antipsychotic drug trifluoperazine (TFP), a dual antagonist of dopamine receptor D2 (DRD2) and CALM (Cook et al., 1994), increases the level of phosphorylated eIF2α. TFP is a classical drug for the treatment of schizophrenia with some side effects including extra pyramidal symptoms such as delayed dyskinesia, lethargy, insomnia, nausea and vomiting, skin and eyes changes, weight gain, etc. (Marques et al., 2004). In this study, we partially elucidate the mechanism by which TFP phosphorylates eIF2α and affects viral replication. We found that TFP significantly restrains VSV and HSV-1 replication in cells by targeting CALM in a PERK-dependent manner and protects mice from lethal VSV attacks. This study sheds light on the development of TFP as a new antiviral drug in the future.

## Materials and methods

### Mice

Six-week-old female wild-type C57BL/6J mice were purchased from Zhejiang Vital River Laboratory Animal Technology Co., Ltd. (Zhejiang, China). During *in vivo* infection studies, 6- to 8-week-old female mice were received 5 mg/kg TFP pretreatment or equal volume phosphate buffered saline (PBS) by intraperitoneal injection twice 36 h (for hour) and 12 h before infected with VSV by tail intravenous (i.v.) injection. TFP was administered 12 h after VSV attack again. The survival condition of mice was recorded and the Kaplan-Meier survival curve was constructed. 18 h after VSV attack, lung tissues from mice were separated for qRT-PCR, hematoxylin-eosin (HE) staining and immunohistochemistry (IHC). H&E staining and IHC was performed by Servicebio Biotechnology (Wuhan, China). For survival experimental studies, mice were intravenously injected with VSV by 4 × 10^6^PFU/g. For organ harvesting, mice were injected with VSV by 2 × 10^6^PFU/g. All animal experiments were undertaken in line with the National Institute of Health Guide for the Care and Use of Laboratory Animals and maintained under SPF (specific-pathogen-free) condition. Cages, bedding, water, and feed were replaced or supplemented regularly. The protocols have been approved by the Animal welfare and Ethics Committee of Sun Yat-sen University Cancer Center (L102012018000Y). All operations were carried out in strict accordance with the operating specifications.

### Cell-lines

Human embryonic kidney 293T (HEK293T) cell lines (from embryonic kidney of female human fetus) and Vero cell lines (from the kidney of a female normal adult African green monkey) were cultured in Dulbecco’s modified Eagle’s medium (DMEM) (Gibco, C11995500BT) supplemented with 10% fetal bovine serum (FBS) (Gibco, 10270-106). A549 human lung adenocarcinoma cell lines (from the lung of a 58 years old male human) and DLD1 human colorectal adenocarcinoma cell lines (from the colorectum of an adult) were cultured in Roswell Park Memorial Institute (RPMI) 1640 medium (Gibco, C11875500BT). HCT116 human colorectal adenocarcinoma cell lines (from the colorectum of a male human) were cultured in McCoy’s 5A medium (KeyGEN, KGM4892N-500). All cell lines were cultured at 37°C under 5% CO2. 293T (#CRL-11268), Vero (#CCL-81), A549 (#CCL-185), DLD1 (#CCL-221), and HCT116 (#CCL-247) were originally obtained from ATCC and all human cell lines were authenticated by China Center for Type Culture Collection (CCTCC). Mycoplasma contamination was routinely checked by PCR analysis and eliminated by treatment with Plasmocin™ (ant-mpt). The primers were as follows: Myco forward 5′-GGG AGC AAA CAG GAT TAG ATA CCC T-3′; Myco reverse 5′-GCA CCA TCT GTC ACT CTG TTA ACC TC-3′.

### Antibodies and reagents

The eIF2α (Phospho-Ser51) Rabbit Monoclonal Antibody (1:1000 for immunoblot and 1:100 for immunohistochemistry, 3398) and eIF2α Rabbit Antibody (1:1000, AF6087) were purchased from Cell Signaling Technology and Affinity, respectively. The Tubulin Mouse Monoclonal Antibody (1:2000, RM2003) was purchased from Ray antibody (Beijing, China). Secondary antibodies were purchased from Jackson (1:5000; Jackson ImmunoResearch Inc.). PAGE Gel Fast Preparation Kit (PG112) was purchased from EpiZyme (Shanghai, China). Protein Marker (M221) was bought from Genstar (Beijing, China). 5 × Dual loading buffer (FD006) and FDbio-Pico ECL (FD8000) were ordered from FDbio science (Hangzhou, China).

### Viruses

Vesicular stomatitis virus-green fluorescent protein (GFP) was provided by Prof. Rongfu Wang (Zhongshan School of Medicine, Sun Yat-sen University, China) and amplified in Vero cells. HSV-1-GFP was provided by Prof. Musheng Zeng (Sun Yat-sen University Cancer Center, China) and amplified in Vero cells. A549, DLD1 or HCT116 cells were infected with VSV (0.1 multiplicity of infection [MOI]) or HSV-1 (10 MOI) for various times, as indicated in the Figures. The virus titer was detected by plaque-forming assays. Before infection with the viruses, Vero cells were covered with 12-well plate until the cells were full. The viruses were diluted ten times from high to low, then Vero cells were infected. Three replicates were made for each concentration, including a blank control group. After 1.5 h of infection, wash with PBS for three times, 2 × DMEM high glucose medium (Genom, GNM12902) and 3% Agar (Sigma, A1296-100G) solution were mixed 1:1 and added to the well plate 1 mL per well. After the mixture solidifies, the 12-well plate was inverted and cultured at 37°C. After 72 h of culture, the green plaque was observed under a fluorescence microscope, and the viral titers of GFP-VSV and GFP-HSV-1 were calculated.

### Drug screening

A549 cells were treated with 27 FDA-approved dopamine receptor antagonists (from Selleck Chemicals, Houston, TX, USA) at a concentration of 15 μM for 12 h. Then, cells were collected to obtain protein and P-eIF2α level was detected. Twenty-seven drugs were as follows: Amisulpride (S1280), Paliperidone (S1724), Quetiapine Fumarate (S1763), Chlorprothixene (S1771), Tetrabenazine (Xenazine) (S1789), Haloperidol (S1920), Pramipexole 2HCl Monohydrate (S2011), Levosulpiride (S2104), Amantadine HCl (S2451), Pramipexole (S2460), Domperidone (S2461), Dopamine HCl (S2529), Benztropine mesylate (S3163), (+, −)-Octopamine HCl (S3188), Ropinirole HCl (S3189), Trifluoperazine 2HCl (S3201), Pergolide Mesylate (S4000), Droperidol (S4096), Penfluridol (S4151), Azaperone (S4219), Rotigotine (S4274), Metoclopramide HCl (S4289), Fluphenazine dihydrochloride (S4569), Fenoldopam mesylate (S4618), Prochlorperazine dimaleate salt (S4631), Brexpiprazole (S4639), Sulpiride (S4655). Tunicamycin was from APExBIO (Houston, USA, #B7417).

### Cell counting Kit-8 assay

Cytotoxicity of TFP was analyzed using a Cell Counting Kit-8 (Beyotime, Nantong, China, #C0037). 5 × 10^3^ A549 cells were seeded in 96-well plates. Different concentrations of TFP or PBS were added and cells were incubated for 36 h. 10 μL CCK-8 solution was added to each well and the cultures were incubated at 37°C for 2 h. Absorbance at 450 nm was measured by a microtiter plate reader. Each group has three replicate wells.

### RNAi

For establishing knockdown cells, shRNAs for *HRI* (shHRI), *PKR* (shPKR), *PERK* (shPERK-1, –2), *GCN2* (shGCN2), *CALM1* (shCALM1-1, –2), or *CALM2* (shCALM2-1, –2) were cloned into lentiviral pLKO.1 construct (Sigma-Aldrich, St. Louis, MO, USA). Lentiviral expressing plasmid, lentiviral packaging plasmid psPAX.2 (Addgene, Cambridge, MA, USA, #12260) and vesicular stomatitis virus-glycoprotein (VSV-G) envelope expressing plasmid pMD2.G (Addgene, Cambridge, MA, USA, #12259) were cotransfected into HEK293T cells. After 48 h, the lentiviruses were used for infecting tumor cells and then screened by puromycin for 3 days. The shRNAs sequences were shown in Supplementary Table 1.

### Immunoblot

Cells were lysed with lysis buffer (50 mM Tris-HCl, pH 7.4, 150 mM NaCl, 0.1% TritonX-100) supplemented with protease inhibitor cocktail (TOPSCIENCE, Shanghai, China, #C0001) and phosphatase inhibitor cocktail I (TOPSCIENCE, Shanghai, China, #C0002) for 30 min. After centrifugation at 14,000 *g* and 4°C for 10 min, supernatants were collected and boiled for 5 min together with 5 × loading buffer (FDbio, FD006) to be used to perform SDS-PAGE. Each sample was loaded with 20–40 μg protein. Proteins were further transferred onto 0.22 μm PVDF membrane (Roche, 3010040001). The membrane was blocked with 5% fat-free milk in Tris-buffered saline added 0.1% Tween-20 (TBS-T) for 1–2 h at room temperature and then incubated with appropriate primary antibody at 4°C overnight. The membrane was washed three times for 10 min each with TBS-T and then incubated with horseradish peroxidase (HRP)-conjugated secondary antibody for 1 h at room temperature. After washing three times with TBS-T, the membrane was flushed with FDbio-Pico ECL (FDbio). ChemiDoc Touch (Bio-Rad) achieved visualization. The uncropped scans of blots are shown in Supplementary Figure 3.

### Quantitative RT-PCR

Total RNA and RNA viruses were extracted with Trizol reagent (Magen, R4801) according to the manufacturer’s instructions. Cells or tissues were rapidly lysed by Trizol reagent, and a three-phase system was formed by chloroform extraction. RNA existed in the aqueous layer. Pure RNA was obtained by precipitation with isopropanol and washing with 75% ethanol. Reverse-transcription products of RNA samples were amplified by the HiScript II Q RT SuperMix for qPCR (Vazyme, R333-01). The reagent contains heat-sensitive DNase, which removes genomic DNA. The products were diluted and used for subsequent RT-PCR. Genomic DNA and DNA viruses were extracted with TIANamp Genomic DNA Kit (TIANGEN, DP304). Adherent cells were treated as cell suspensions to obtain cells. Cells were lysed in a specific buffer system adding RNaseA (TIANGEN, RT405) and proteinase K. After incubation for 70°C 10 min, absolute ethanol was added. The solution and flocculent precipitate were transferred to the centrifugal column. Genomic DNA was selectively adsorbed on the silicon matrix membrane in the centrifugal column under a high salt state. Then, genomic DNA was obtained by rapid rinsing, centrifugation and elution. Real-time PCR with SYBR Green was performed by Hieff qPCR SYBR Green Master Mix (YEASEN, 11201ES08*). Primers used were shown in Supplementary Table 2. All target gene expression or DNA level was normalized to the control gene encoding *18S* (for human cell lines) or *Actb* (β*-Actin*, for mouse tissues) in each sample and the 2^–ΔΔCt^ method (Livak and Schmittgen, 2001) was used to calculate relative expression changes under the assumption that the amplification efficiency of target genes and reference genes is 100%.

### Statistical analysis

The data were analyzed with SPSS software and generated using GraphPad Prism 8. For two independent groups, Student’s *t*-test was used to determine statistical significance. For three and more independent groups, we used one-way ANOVA with Bonferroni’s post-test. The data used for the *t*-test and the one-way ANOVA were considered to be normally distributed by passing the Shapiro–Wilk normality test (Shapiro and Wilk, 1965). Statistical details for individual experiments can be found in the Figure legends. The data on the expression of CALM and DRD2 in tumor tissues and normal tissues are derived from http://gemini.cancer-pku.cn/. Survival curves were analyzed using the Kaplan–Meier method and the logrank test. Statistical significance was two-tailed and *p* < 0.05 was considered statistically significant *P*-values indicated by asterisks in the Figures as followed: *p < 0.05, ^**^p < 0.01, ^***^p < 0.001, and n.s. = non-significant.

## Results

### Pharmaceutical screening identified trifluoperazine as an eIF2α phosphorylation inducer

**FIGURE 1:**
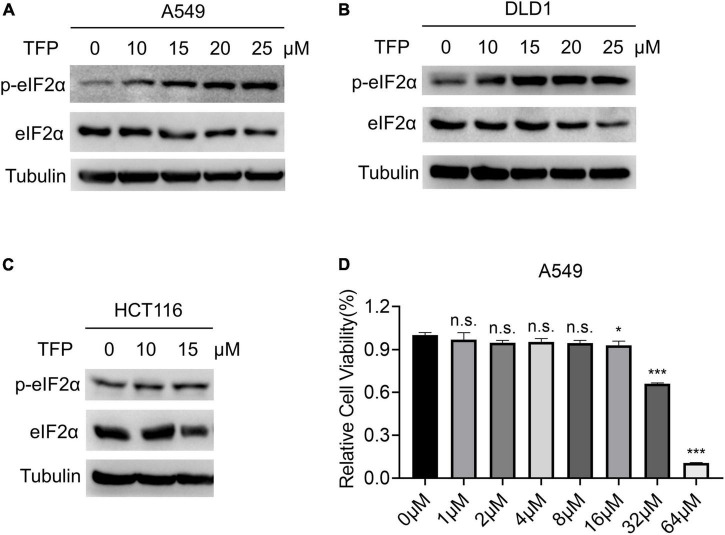
TFP promotes the phosphorylation of eIF2α. **(A–C)** A549, DLD1, and HCT116 cells were treated with TFP at different concentrations from low to high for 12 h. **(D)** A549 cells were treated with different concentrations of TFP for 36 h, and cell viability was detected by the Cell Counting Kit-8 (CCK-8) assay. Statistical analysis was performed by one-way ANOVA with Bonferroni’s post-test, comparing each group with the control group. *p < 0.05, ***p < 0.001, n.s., not significant.

To identify new effective antiviral drugs, we screened FDA approved drug library in human lung adenocarcinoma A549 cells. Because of the importance of eIF2α in translation and the dependence of the viruses on the host protein synthesis system, we evaluated eIF2α phosphorylation (P-eIF2α) level in A549 cells after drug treatment by western blots. We found that TFP, a dual DRD2/CALM antagonist, increased the ratio of P-eIF2α/eIF2α by nearly three times compared with the mock group 12 h after treatment, while the other 26 dopamine receptor agonists/antagonists showed no significant change (Supplementary Figures 1A,B). With the increase in TFP concentration, P-eIF2α level elevated and exhibited a concentration-dependent effect under experimental conditions (Figure 1A; Supplementary Figure 1C). We used tunicamycin (Tm), an agent that causes endoplasmic reticulum (ER) stress by inhibiting protein glycosylation as a positive control (Novoa et al., 2001). Treatment of Tm for 2 h and 4 h promoted the phosphorylation of eIF2α, which is consistent with a previous report (Tsaytler et al., 2011; Supplementary Figure 1C). We confirmed this phenomenon in TFP-treated DLD1 (Figure 1B) and HCT116 cells (Figure 1C). Because a high concentration of TFP caused cell death (Figure 1D) and the half-life of TFP is about 12.5 h (Midha et al., 1983), we chose 15 μM and 12 h as an optimal condition of TFP treatment in the subsequent experiments. From the above results, it is consistently shown that TFP promotes the phosphorylation of eIF2α.

### Trifluoperazine and its target calmodulin influence viral replication

**FIGURE 2:**
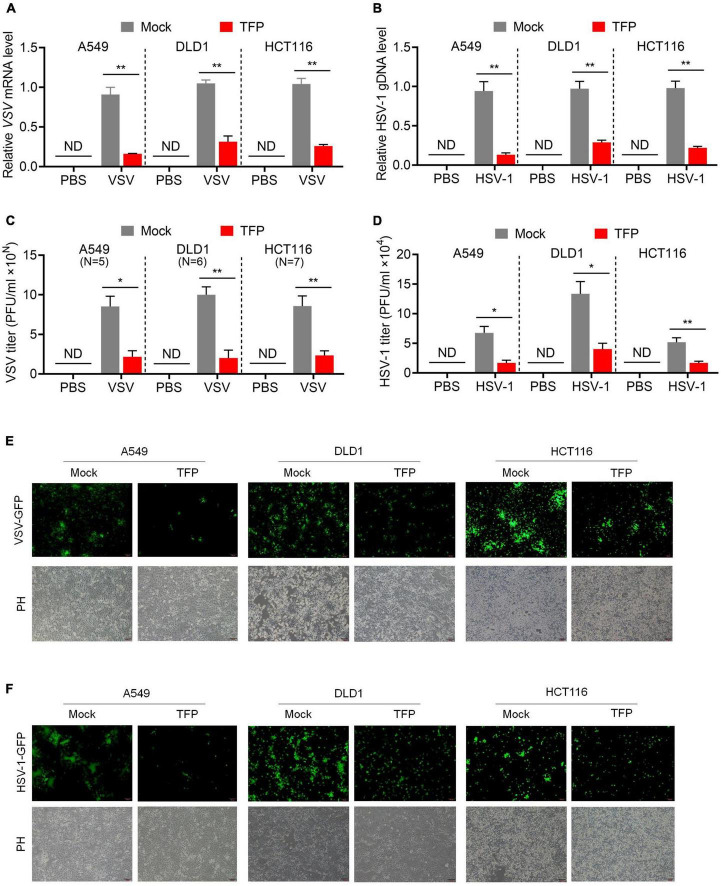
TFP inhibits VSV and HSV-1 replication. **(A,B)** qPCR analysis of VSV mRNA expression **(A)** and HSV-1 gDNA expression **(B)** in mock or TFP-treated A549, DLD1, and HCT116 cells. Cells were infected with VSV-GFP (MOI = 0.1) for 12 h or HSV-1 (MOI = 10) for 24 h after 12 h of 15 μM TFP treatment. **(C,D)** Plaque assay of VSV titers **(C)** and HSV-1 titers **(D)** in mock or TFP-treated A549, DLD1, and HCT116 cells shown in panels **(A,B)**. The “10^N^” of the Y-axis in panel **(C)** means the magnitude of VSV titer of A549, DLD1, and HCT116 cells are 10^5^, 10^6^, and 10^7^, respectively. **(E,F)** Images of green fluorescence change under a fluorescence microscope in mock or TFP-treated A549, DLD1, and HCT116 cells shown in panels **(A,B)**. Scale bars, 100 μm. ND, not detected. *p < 0.05, **p < 0.01 (two-tailed Student’s *t*-test). Data from three independent experiments [means ± SDs of triplicate assays **(A–D)**] or representative data from three independent experiments with similar results **(E,F)** are shown.

Then we tested whether TFP affects viral replication. We chose RNA virus VSV and DNA virus HSV-1 to verify our presumption, in the usage of the simplicity and convenience of the experimental assays of these viruses. After TFP pretreatment for 12 h, we infected A549, DLD1 or HCT116 cells with GFP-encoded VSV (VSV-GFP) or HSV-1 (HSV-1-GFP), respectively, and compared viral replication in mock and treated cells. The results of qPCR showed that the level of VSV mRNA and HSV-1 gDNA decreased significantly in TFP pretreatment cells (Figures 2A,B). Consistent with these results, plaque assays revealed a lower virus titer in drug-treated cells with about three to four folds (Figures 2C,D). We also observed decreased green fluorescence under a fluorescence microscope (Figures 2E,F). Bright-field photomicrographs presented that 15 μM TFP treatment had no obvious effect on cell growth, indicating that these results are credible (Figures 2E,F). These results suggest that TFP inhibits VSV and HSV-1 replication effectively through increasing the phosphorylation level of eIF2α.

**FIGURE 3:**
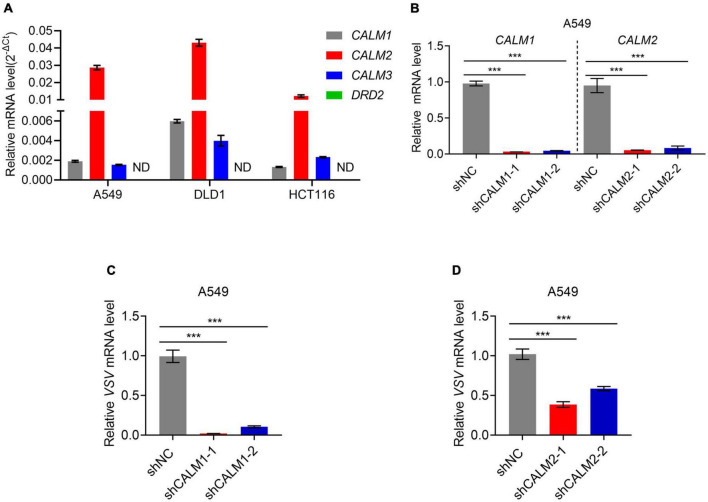
Calmodulin is involved in the regulation of PERK-eIF2α. **(A)** qPCR analysis of *CALM1*, *CALM2*, *CALM3*, and *DRD2* expression in A549, DLD1, and HCT116 cells. **(B)** qPCR analysis of *CALM1* and *CALM2* knockdown efficiency in A549 cells. **(C)** qPCR analysis of VSV mRNA expression of shNC, shCALM1-1, and shCALM1-2 A549 cells infected with VSV-GFP (MOI = 0.1) for 12 h. **(D)** qPCR analysis of VSV mRNA expression of shNC, shCALM2-1, and shCALM2-2 A549 cells infected with VSV-GFP (MOI = 0.1) for 12 h. ***p < 0.001 (one-way ANOVA with Bonferroni’s post-test). Data from three independent experiments [means ± SDs of triplicate assays **(A–D)**] are shown.

As a dual DRD2/CALM antagonist, TFP used to be a commonly prescribed antipsychotic medication. To figure out which is the target of TFP in antiviral response, we searched for *CALMs* and *DRD2* gene expression in various normal tissues in GE-mini database. *DRD2* expression level is extremely low in non-nervous tissues, contrary to the universal expression of three genes encoding calmodulin (*CALM1*, *CALM2*, and *CALM3*) in eukaryotic cells (Supplementary Figure 2A). We also tested *DRD2* and *CALMs* expression in A549, DLD1, and HCT116 cells. As expected, qPCR results showed that the expression level of *CALM1/2/3* was much higher than *DRD2*, which was almost undetectable (Figure 3A). We reasonably assumed that the antiviral activity of TFP is dependent on CALM, but not DRD2. The amino acid sequences of the three members of the CALM family are very similar, among which CALM1 and CALM3 differ by only one amino acid (Supplementary Figure 2B). Therefore, we designed shRNAs to construct *CALM1* and *CALM2* knockdown cell lines (Figure 3B) and infected cells with VSV. The results showed that the replication of VSV decreased significantly after the knockdown of *CALM1* or *CALM2* (Figures 3C,D), suggesting that CALM but not DRD2 is involved in the antiviral activity of TFP.

### Trifluoperazine restrains viral replication in a PERK-dependent manner

**FIGURE 4:**
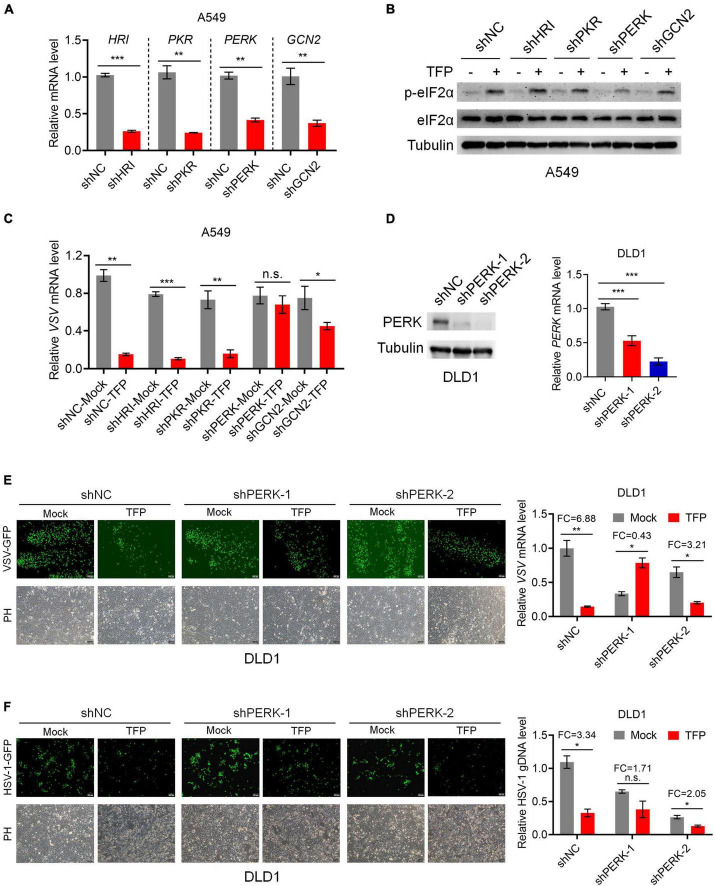
TFP phosphorylates eIF2α through activating PERK. **(A)** qPCR analysis of *HRI*, *PKR*, *PERK*, and *GCN2* knockdown efficiency in A549 cells. **(B)** Immunoblot analysis of P-eIF2α and eIF2α levels of shNC, shHRI, shPKR, shPERK, and shGCN2 A549 cells treated with 15 μM TFP for 12 h. **(C)** qPCR analysis of VSV mRNA expression in the cells shown in panel **(B)**. **(D)** Immunoblot and qPCR analysis of *PERK* knockdown efficiency in DLD1 cells. **(E)** shNC, shPERK-1, and shPERK-2 DLD1 cells were treated with 15 μM TFP for 12 h before infected with VSV-GFP (MOI = 0.1) for another 12 h. Viral replication was detected using fluorescence microscopy and qPCR. **(F)** shNC, shPERK-1, and shPERK-2 DLD1 cells were treated with 15 μM TFP for 12 h before infection with HSV-1 (MOI = 10) for 24 h. Viral replication was detected using fluorescence microscopy and qPCR. Scale bars, 100 μm. ND, not detected. *p < 0.05, **p < 0.01, ***p < 0.001, n.s., not significant [two-tailed Student’s *t*-test (**A,C,E,F**, right)] or one-way ANOVA with Bonferroni’s post-test (**D**, right). Data from three independent experiments [means ± SDs of triplicate assays (**A,C,D–F**, right)] or representative data from three independent experiments with similar results (**B,D–F**, left) are shown.

According to the abovementioned, the phosphorylation of eIF2α reduces the transfer of TC to the ribosome and decreases the global translation eventually. There are four upstream kinases that mediate the phosphorylation of eIF2α, PKR, PERK, GCN2, and HRI. To elucidate the mechanisms by which TFP increases P-eIF2α level, we designed shRNA that separately targeted the four kinases to construct knockdown cell lines (Figure 4A). Western blot showed the level of P-eIF2α reduced after knockdown of PERK, and qPCR results indicated that the VSV mRNA was not influenced in the presence of TFP, in comparison, knockdown of other three kinases showed no impact on the antiviral effect of TFP (Figures 4B,C). Then, we generated PERK stable knockdown DLD1 cells with two different shRNAs for further study (Figure 4D). We confirmed that in the PERK-knockdown DLD1 cell lines, the antiviral effect of TFP on the replication of VSV and HSV-1 was attenuated, while there was no impact on cell growth (Figures 4E,F). Based on these results, TFP stimulates eIF2α phosphorylation through activating PERK, thus repressing viral replication.

### Trifluoperazine protects mice from lethal vesicular stomatitis virus attack

**FIGURE 5:**
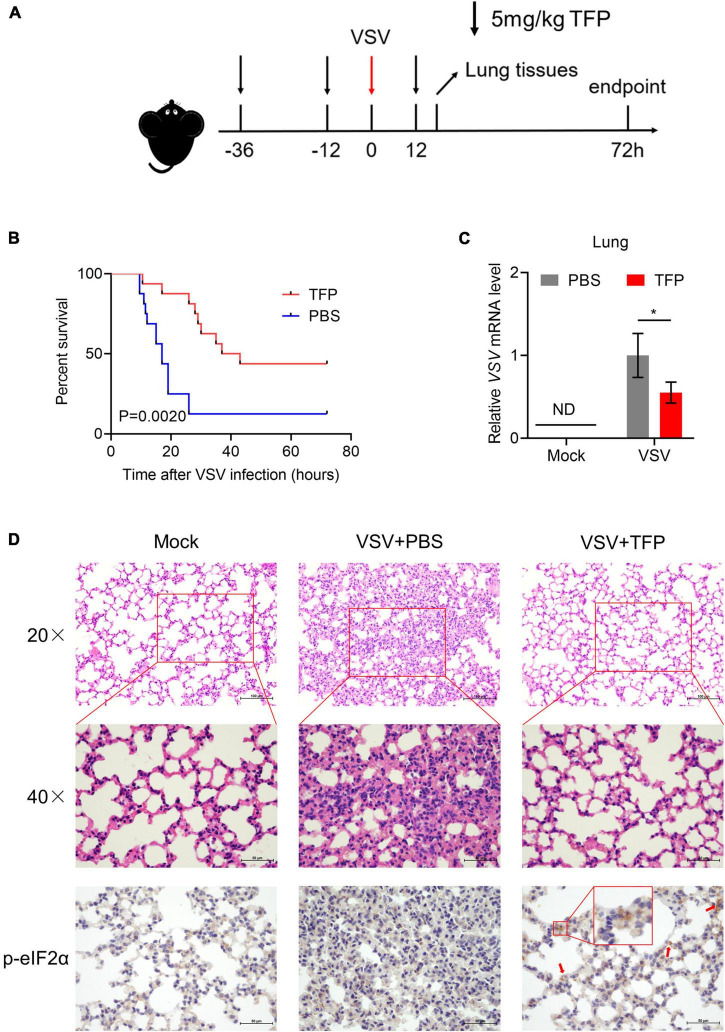
TFP protects mice from lethal VSV attack. **(A)** Schematic of VSV attack model in C57BL/6J mice with TFP treatment. Hours are indexed based on the time of VSV i.v. injection. Mice received 5 mg/kg TFP or equal volume PBS twice before VSV infection by intraperitoneal injection. TFP was administered 12 h after the VSV attack again. Lung tissues were harvested 18 h after the VSV attack. For survival experimental studies, the observation endpoint was 72 h. **(B)** Survival curves (*n* = 16 mice per group, Log-rank test) of 6- to 8-week-old C57BL/6J mice infected with VSV (4 × 10^6^PFU/g) *via* tail i.v. injection. **(C)** qPCR analysis of relative VSV mRNA level in lung from mice corresponding to panel **(A)**. **(D)** Images of H&E staining and IHC of lung sections from the mice shown in panel **(A)**. Scale bars, 50 μm or 100 μm. *p < 0.05 (one-tailed Student’s *t*-test). Data from three independent experiments [means ± SDs of triplicate assays **(C)**] or representative data from three independent experiments with similar results **(D)** are shown.

**FIGURE 6:**
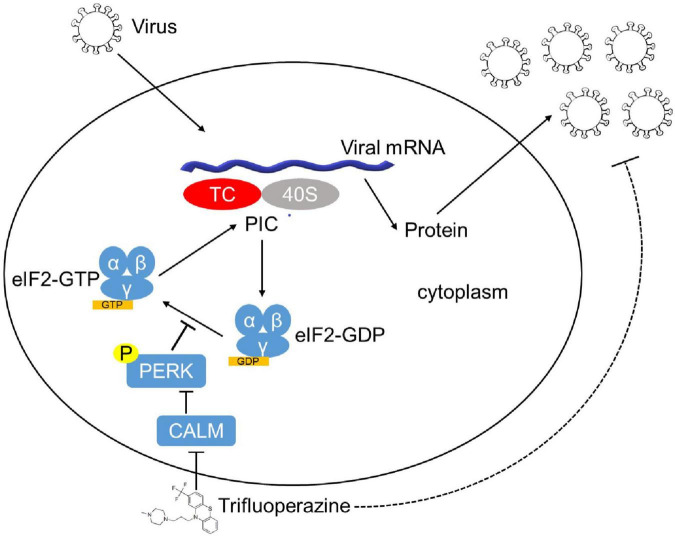
The model of the mechanism of TFP inhibiting viral replication. After infection, the virus synthesizes proteins in a host cell dependent manner. eIF2 (α, β, and γ), a key factor involved in translation initiation, functions to form eIF2–GTP–Met-tRNA_i_^Met^ TC that binds to 40S subunit. Phosphorylation of eIF2α mediated by HRI, PKR, PERK, and GCN2 inhibits the conversion from eIF2⋅GDP to the active form eIF2⋅GTP and reduces TC delivered to the ribosome, resulting in global translation attenuation. FDA-approved drug TFP promotes eIF2α phosphorylation by targeting CALM in a PERK-dependent manner, which decelerates protein translation and restrains the replication of both RNA and DNA viruses.

Finally, we assessed the antiviral activity of TFP *in vivo* using 6- to 8-week-old C57BL/6J mice. 5 mg/kg TFP or equal volume PBS were administrated 36 h and 12 h before and 12 h after infected with VSV by tail intravenous (i.v.) injection (Figure 5A). Survival curves showed a significant difference in mice between TFP and PBS groups after being challenged with a lethal dose of VSV infection (Figure 5B). More than half of the mice from the PBS group died within 24 h while the mortality of mice in the TFP group was less than one in five (Figure 5B). Until the endpoint of observation, the survival rate of the TFP group was nearly 50% (7/16) and that of the PBS group was only 12.5% (2/16). Consistently, the VSV mRNA abundance in lung tissues was notably reduced in mice treated with TFP (Figure 5C). H&E staining of the lungs showed that VSV infection induced the infiltration of a greater number of immune cells and much more severe tissue injury in mice not treated with TFP (Figure 5D). The lung tissue structure of mice from the TFP group was more clear and more complete, with a higher level of P-eIF2α detected by IHC (Figure 5D). Based on these data, TFP represses virus replication through activating PERK and phosphorylating eIF2α *in vitro* and *in vivo*, which depends on one of its target CALM (Figure 6), protecting mice from lethal VSV attacks.

## Discussion

The nascent peptides are transferred from cytosol to the ER lumen in an unfolded state and undergo molecular chaperone-assisted folding to acquire their appropriate three-dimensional conformation, together with complex modifications. Adequate folding and post-translational modification of proteins is essential for normal function. Unlike DNA replication, transcription and translation, protein folding is an error-prone process (Hetz et al., 2020). Misfolded and aggregated proteins cause ER stress and cell death, leading to some diseases such as neurodegeneration, genetic and developmental disorders, and may also promote the initiation and progression of cancer (Wang and Kaufman, 2014, 2016). ER stress triggers unfolded protein response (UPR), which is activated by the coordinated action of three ER transmembrane stress sensors inositol-requiring protein 1 (IRE1α), PERK, and activating transcription factor 6α (ATF6α) in mammals. Under homeostatic conditions, the luminal domains of these ER stress sensors bind to BiP (binding immuno-globulin protein, also known as GRP78 and HSPA5) and remain inactive. When unfolded proteins accumulate in the ER lumen, BiP will separate from these sensors because of its higher affinity for unfolded proteins, resulting in the aggregation of these transmembrane signal proteins and transmitting UPR signals to downstream (Grootjans et al., 2016). In this process, PERK undergoes oligomerization and induces autophosphorylation to activate the kinase domain. P-PERK phosphorylates and inactivates eIF2α, decelerating protein translation to restore proteostasis.

In this study, we found that FDA-approved antipsychotic TFP increases eIF2α phosphorylation by activating PERK to inhibit the replication of RNA viruses and DNA viruses. As PERK is one of the three sensors of UPR, TFP may affect protein folding in the ER, which is very sensitive to environmental changes, such as changes in Ca^2+^ levels, redox status, nutritional status, increased protein synthesis rate, pathogens or inflammatory stimuli. These changes can cause protein folding interruption, leading to unfolded or misfolded protein accumulation and inducing UPR (Wang and Kaufman, 2014). Maintaining the Ca^2+^ homeostasis of the ER is of great importance to correct protein folding, and the disturbance of Ca^2+^ homeostasis leads to the accumulation of misfolded proteins and ER stress (Carreras-Sureda et al., 2018). CALM, the target of TFP, is a Ca^2+^ sensor, which detects and responds to a biologically relevant range of intracellular free Ca^2+^ concentration (Chin and Means, 2000). TFP binds to CALM in a Ca^2+^-dependent manner (Levin and Weiss, 1977), which induces tertiary structure change of CALM from an elongated dumb-bell, with exposed hydrophobic surfaces, to a compact spherical structure that no longer interacts with its target enzymes (Vandonselaar et al., 1994; Matsushima et al., 2000). It has been reported that the binding of TFP and CALM changes the affinity of CALM for Ca^2+^ (Tanokura and Yamada, 1985; Feldkamp et al., 2010). For example, TFP inhibits CALM-sensitive Ca^2+^-ATPase in human and rat red blood cells (Luthra, 1982); TFP inhibits Ca^2+^-dependent secretion in adrenal medulla cells by inhibiting Ca^2+^ absorption (Wada et al., 1983). It is reasonable to speculate that the binding of TFP to CALM not only changes the affinity of CALM for Ca^2+^, but also changes the Ca^2+^ concentration in cells, which consequently destroying the ER Ca^2+^ homeostasis. In addition, BiP can also bind Ca^2+^, and changes in Ca^2+^ concentration may affect the function of BiP in protein folding (Suzuki et al., 1991; Lamb et al., 2006). In summary, TFP possibly disrupts ER Ca^2+^ homeostasis by changing the intracellular and ER Ca^2+^ level, thereby increasing unfolded or misfolded proteins, activating PERK, and inducing UPR and ER stress.

Virus-related diseases are seriously threatening human health, such as coronavirus disease 2019 (COVID-19) caused by SARS-CoV-2, influenza, HBV-related liver cancer HPV-related cervical cancer, and so on (Krammer et al., 2018; Krump and You, 2018; Lamers and Haagmans, 2022). The strategies against viral diseases are prevention (vaccines) and treatment (antiviral drugs and antibodies). However, there are currently only 10 viruses with clinically approved antiviral drugs available among more than 220 known viruses that infect humans. The time required to develop a vaccine is very long, usually between 3 and 10 years, and not all vaccines that induce protection against infection are successful (Adamson et al., 2021). It is urgent to develop new and effective antiviral drugs. Most approved antiviral drugs target viral enzymes that play a key role in viral replication. Viral polymerases are very effective drug targets. Polymerase inhibitors can be divided into nucleoside analogues and non-nucleoside allosteric inhibitors. Such antiviral drugs have a wide range of effects, for example, nucleoside analogue remdesivir can be used to treat a variety of RNA virus infections (Liang et al., 2020). Protease inhibitors are another class of approved major antiviral drugs, mainly used to treat HIV and HCV infection, such as saquinavir. Drugs targeting other viral enzymes tend to be specific, such as influenza neuraminidase (NA) inhibitors oseltamivir. Entry/fusion inhibitors target non-enzymatic viral processes (De Clercq and Li, 2016).

In addition to antiviral drugs that directly target host-virus interactions, interferons, immunostimulators, oligonucleotides, and antimitotic inhibitors have also been used for antiviral therapy (De Clercq and Li, 2016). There are few reports on the antiviral function of drug targeting PERK-eIF2α axis. As mentioned, a small molecule inhibitor salubrinal blocks HSV protein-mediated dephosphorylation of eIF2α and inhibits virus replication (Boyce et al., 2005). Montelukast (MK), a drug for treating asthma, induces PERK phosphorylation and stimulates UPR, thus reducing virus multiplication (Landeras-Bueno et al., 2016). Two FDA-approved thiopurine drugs, 6-thioguanine (6-TG) and 6-thioguanosine (6-TGo) also induce the UPR and prevent influenza virus replication by impeding viral glycoprotein accumulation (Slaine et al., 2021). In this study, we found that the FDA-approved drug TFP inhibits the replication of RNA viruses and DNA viruses probably through disrupting ER Ca^2+^ homeostasis and inducing UPR, resulting in decelerating protein translation. However, this study has some limitations. The specific mechanism of TFP on inducing the phosphorylation of eIF2α was not fully elucidated. In addition, more virus strains are needed to evaluate the antiviral function of TFP in animal experiments. This study indicates that TFP targets host protein, suggesting that it is likely to have a broad-spectrum antiviral function and is worthwhile to conduct relevant clinical trials for further exploration.

## Conclusion

In this study, we found that the FDA-approved drug trifluoperazine (TFP) inhibits the replication of RNA and DNA viruses, probably through disturbing the phosphorylation homeostasis of the PERK-eIF2α axis, resulting in decelerating protein translation. TFP targets host proteins, suggesting that TFP inhibits virus replication through broad-spectrum mechanisms, which is worth further evaluation in clinical trials in the future.

## Data availability statement

The raw data of this study have been deposited in Research Data Deposit database (https://www.researchdata.org.cn/), with the Approval Number as RDDB2022872977.

## Ethics statement

The animal study was reviewed and approved by Animal Welfare and Ethics Committee of Sun Yat-sen University Cancer Center.

## Author contributions

YM and ZW performed most of the experiments and analyses. YM, ZW, and CY performed the mouse experiments. QZ, WC, and SZ provided technical assistance. SC and CS conceived the study. SC and YM wrote the manuscript. All authors contributed to the article and approved the submitted version.
