# Supplementary material for: The Food and Drug Administration-approved antipsychotic drug trifluoperazine, a calmodulin antagonist, inhibits viral replication through PERK-eIF2α axis
Source: Front Microbiol. 2022 Nov 1;13:979904. doi: 10.3389/fmicb.2022.979904 (PMC9664084; doi:10.3389/fmicb.2022.979904)
Supplement: Supplementary file 1 [file Data_Sheet_1.docx]

***Supplementary Materials***

**The Food and Drug Administration-approved antipsychotic drug trifluoperazine, a calmodulin antagonist, inhibits viral replication through PERK-eIF2α axis**

Yizhi Mao, Ziyang Wang, Chen Yao, Qi Zeng, Wei Cheng, Shimeng Zhang, Shuai Chen, Chunjie Sheng

**SUPPLEMENTARY FIGURES**


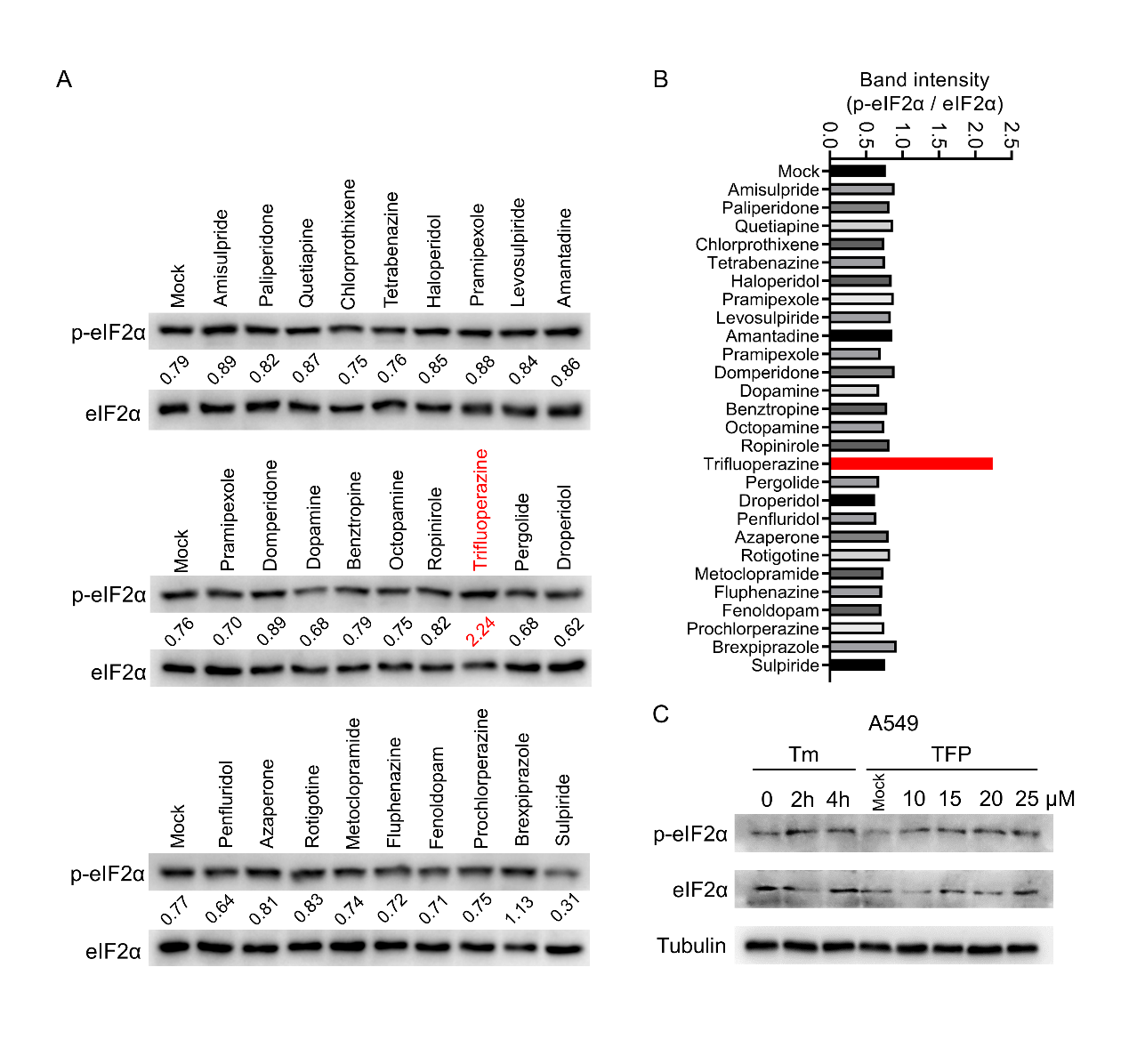


**Figure S1.** Drug screening indicates that TFP promotes the phosphorylation of eIF2α. (A) A549 cells were treated with 27 DRD2 antagonists at a concentration of 15μM for 12 h. Phosphorylation of eIF2α and eIF2α were detected by immunoblot. (B) Histogram of the ratio of P-eIF2α / eIF2α shown in (A). (C) A549 cells were treated with tunicamycin (1μg/ml) for the indicated times and TFP at different concentration for 12 h.

A


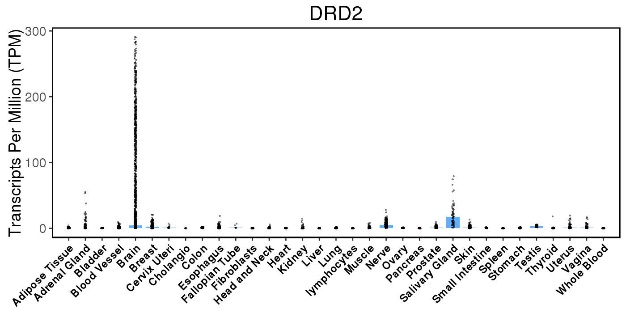

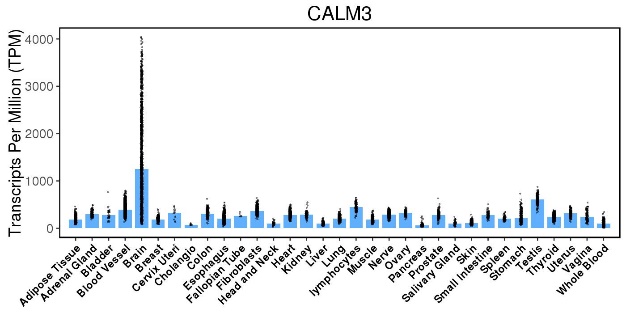

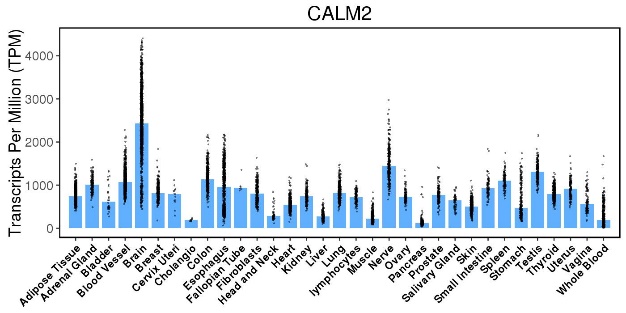

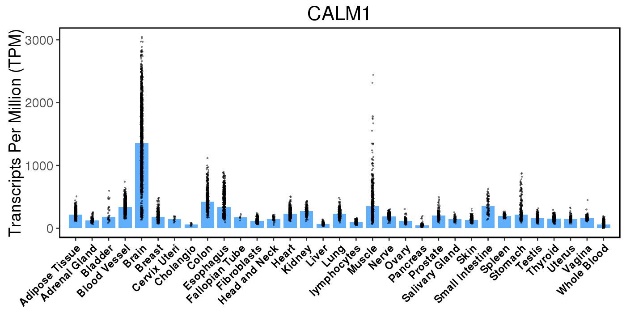


B


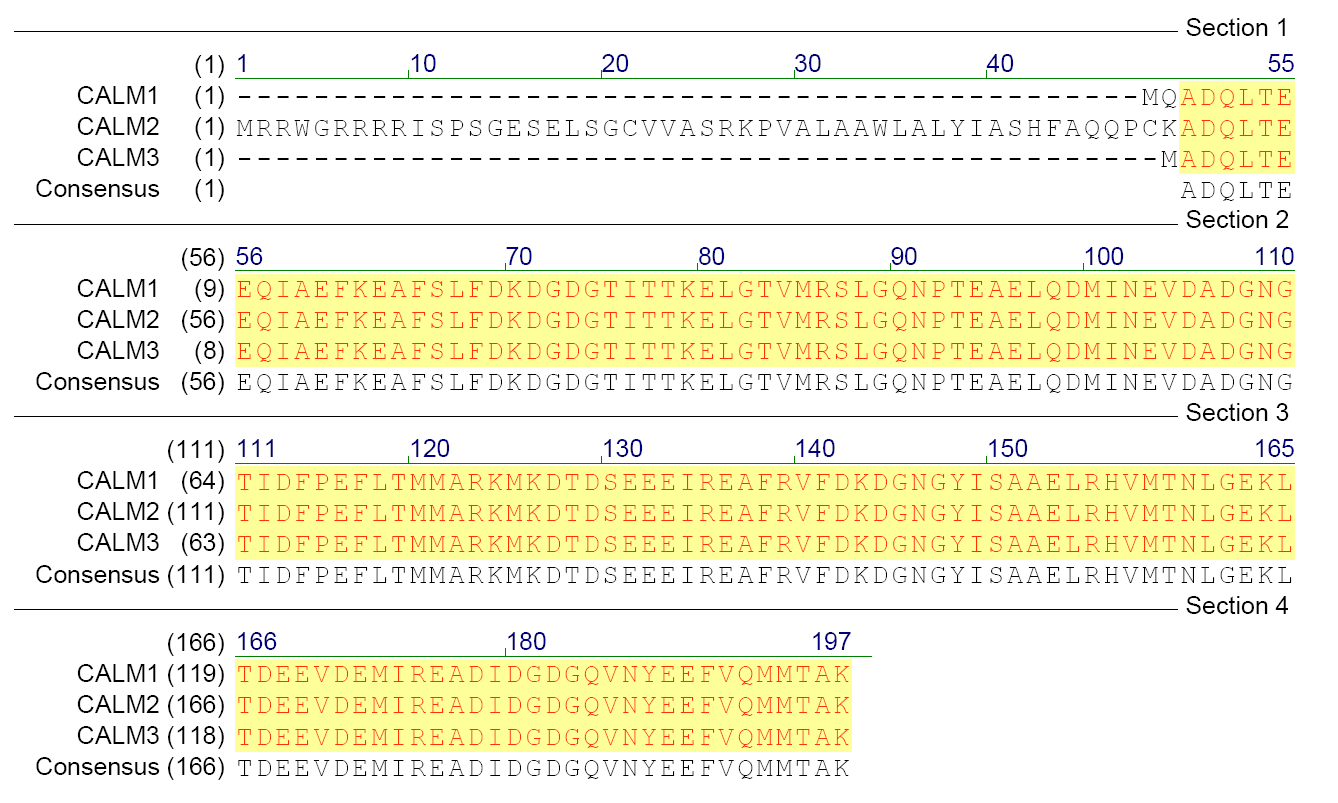


**Figure S2.** (A) CALM1, CALM2, CALM3 and DRD2 expression profile in various normal tissues as identified through the GE-mini website. (B) Multiple sequence alignments of CALM1, CALM2 and CALM3.


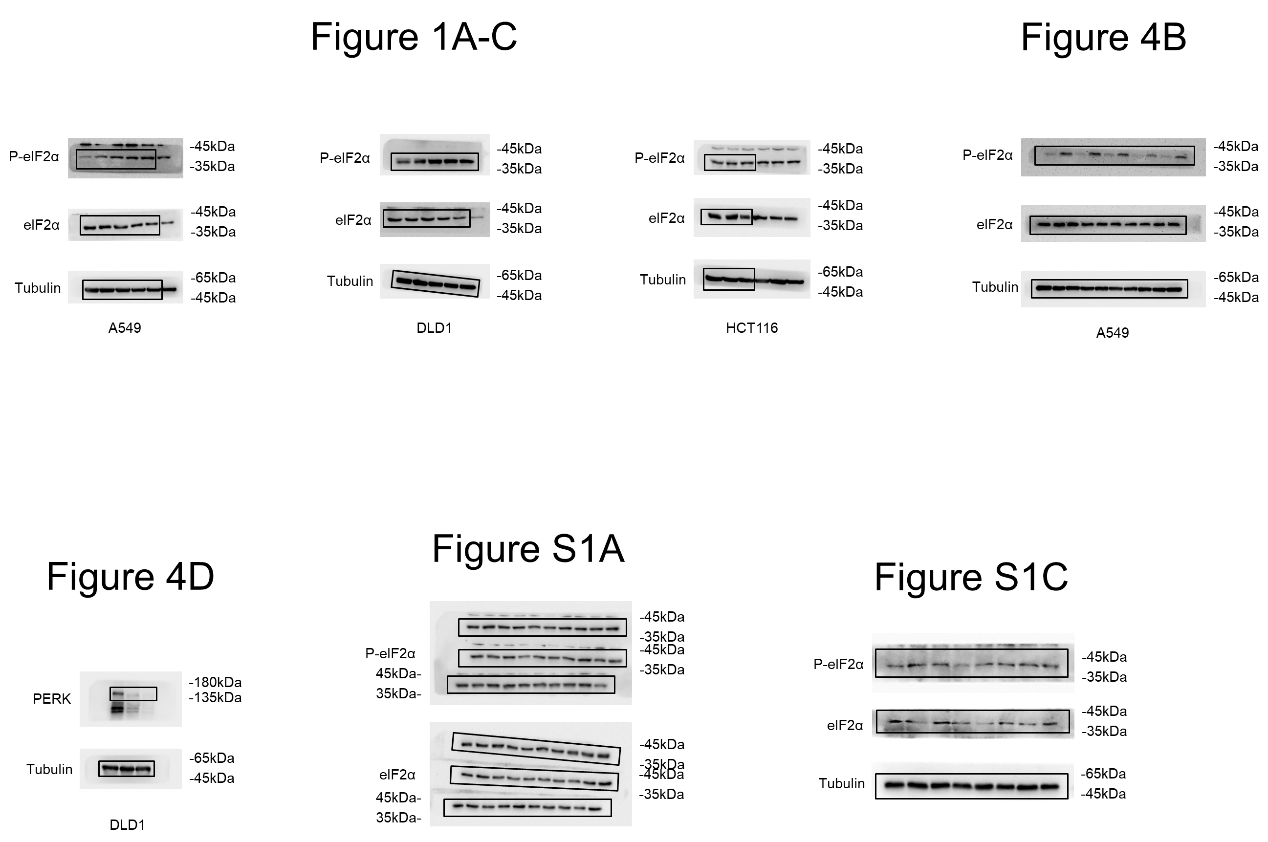


**Figure S3.** The uncropped scans of blots.

**SUPPLEMENTARY TABLES**

Table S1. shRNAs sequences for RNAi. Related to MATERIALS AND METHODS. Sequences are listed 5’ to 3’.

| shHRI | CCGGAGCTACTTTGCCAGACGTTTACTCGAGTAAACGTCTGGCAAAGTAGCTTTTTTG |
| --- | --- |
| shPKR | CCGGGCTGAACTTCTTCATGTATGTCTCGAGACATACATGAAGAAGTTCAGCTTTTTG |
| shPERK-1 | CCGGTAGCAGCAATCCCTAATATATCTCGAGATATATTAGGGATTGCTGCTATTTTTG |
| shPERK-2 | CCGGGCATCTGCCTGGTTACTTAAGCTCGAGCTTAAGTAACCAGGCAGATGCTTTTTG |
| shGCN2 | CCGGCTAGGCGAGAACGTCAGTATTCTCGAGAATACTGACGTTCTCGCCTAGTTTTTG |
| shCALM1-1 | CCGGGACGGACAAGTCAACTATGAACTCGAGTTCATAGTTGACTTGTCCGTCTTTTTG |
| shCALM1-2 | CCGGAGAAGCTGAATTGCAGGATATCTCGAGATATCCTGCAATTCAGCTTCTTTTTTG |
| shCALM2-1 | CCGGGGACAGTCAACAATATGTACTCTCGAGAGTACATATTGTTGACTGTCCTTTTTG |
| shCALM2-2 | CCGGGGACTTCATTCCTCCATGTTTCTCGAGAAACATGGAGGAATGAAGTCCTTTTTG |

Table S2. Primers for mRNA Quantification. Related to MATERIALS AND METHODS. Primers are listed 5’ to 3’.

| VSV forward | ACGGCGTACTTCCAGATGG |
| --- | --- |
| VSV reverse | CTCGGTTCAAGATCCAGGT |
| HSV-1 forward | TGGGACACATGCCTTCTTGG |
| HSV-1 reverse | ACCCTTAGTCAGACTCTGTTACTTACCC |
| *HRI* forward | ACCCCGAATATGACGAATCTGA |
| *HRI* reverse | CAAGTGCTCCAGCAAAGAAAC |
| *PKR* forward | TGGAAAGCGAACAAGGAGTAAG |
| *PKR* reverse | CCAAAGCGTAGAGGTCCACTT |
| *PERK* forward | GGAAACGAGAGCCGGATTTATT |
| *PERK* reverse | ACTATGTCCATTATGGCAGCTTC |
| *GCN2* forward | AAATGCCCACCTACCTATCCA |
| *GCN2* reverse | CCTCCCCACAGTGTTTCTTGG |
| *CALM1* forward | TTGACTTCCCCGAATTTTTGACT |
| *CALM1* reverse | GGAATGCCTCACGGATTTCTT |
| *CALM2* forward | TGCATGTGGCTTACTCTGGA |
| *CALM2* reverse | ACTGTCCATAGTCCACGCAG |
| *CALM3* forward | GTGGGGATGGTCGCTTTGTA |
| *CALM3* reverse | TCCCTCTCCCCACCCTTTAG |
| *18S* forward | GTAACCCGTTGAACCCCATT |
| *18S* reverse | CCATCCAATCGGTAGTAGCG |
| *Actb* forward | AGTGTGACGTTGACATCCGT |
| *Actb* reverse | GCAGCTCAGTAACAGTCCGC |
